# Perspectives on Continuing Education Programs for Foundation-Level Drugstore Pharmacists in Japan

**DOI:** 10.3390/pharmacy8040223

**Published:** 2020-11-19

**Authors:** Tomoko Terajima, Kumiko Matsushita, Seiichiro Yamada, Hiroaki Suzuki, Shingo Yano, Mizue Makimura, Shigeo Yamamura

**Affiliations:** 1 Department of Biostatistics, Faculty of Pharmaceutical Sciences, Josai International University, Togane 283-8555, Japan; terajima@jiu.ac.jp; 2 Secretariat, AEON HAPYCOM Comprehensive Training Organization, Chiba 261-8515, Japan; matsushita-kumiko6@aeonpeople.biz (K.M.); tcr.slr1@gmail.com (S.Y.); hiro_suzuki@aeonpeople.biz (H.S.); 3 Boards of Certification, AEON HAPYCOM Comprehensive Training Organization, Chiba 261-8515, Japan; y-shingo@wh2.fiberbit.net (S.Y.); mizuem01@yahoo.co.jp (M.M.)

**Keywords:** continuing education, continuing professional development, foundation-level pharmacist, drugstore pharmacists

## Abstract

Background: Continuing education (CE) is important for developing and updating pharmacists’ knowledge, skills, and attitudes. CE programs should be developed according to social requirements but also based on personal requirements depending on the sectors the pharmacists work in. This research aims to explore perspectives on CE programs for foundation-level drugstore pharmacists in Japan. Method: Foundation-level drugstore pharmacists were asked what CE programs or training they needed to develop patient care or customer satisfaction. Results: We obtained 417 opinions (multiple answers were allowed) in 280 responses from 460 pharmacists (male: 245 and female: 215). The products and goods about which drugstore pharmacists wanted to learn covered a wide range. They wanted to learn about taping skills, tests, and products and devices related to care of the elderly. Taping skill would be quite unique for drugstore pharmacists. For special populations, they wanted knowledge and skills related to pregnancy tests and the safe use of medication by pregnant or lactating women. Conclusion: Drugstore pharmacists in Japan have different CE and continuing professional development (CPD) requirements from community pharmacists. The benefits of CE programs meeting pharmacists’ requirements should be evaluated in future research.

## 1. Introduction

Pharmacists as healthcare professionals should strive to maintain their competence in order to deliver the best quality of service to patients or customers. Continuing education (CE) or continuing professional development (CPD) is necessary to develop and update their professional skills. CPD is a lifelong learning that is applied in practice [1].

In some countries, pharmacists must take a CE program and register the required points, units, or credits for a particular period in order to be able to continue working as a registered pharmacist. In the USA, it depends on the state, and CE programs or courses must satisfy continuing education requirements [2], while in Canada, all CE programs are accredited by the Canadian Council on Continuing Education in Pharmacy [3]. There are many reports on the current situation of CE and CPD in other countries [4,5,6].

In Japan, the Japan Pharmacy Education Center (JPEC) oversees the certification of qualified pharmacists and the publication of training manuals and teaching materials [7]. The Japan Pharmaceutical Association (JPA) launched the JPA Lifelong Learning Support System (JPALS) for CPD in April 2012, a support system that encourages lifelong learning for community pharmacists [8].

A study of the competency framework of foundation-level Japanese pharmacists identified various groupings of pharmaceutical public health and pharmaceutical care issues relevant to their professional development [9] as defined in the International Pharmaceutical Federation (FIP) Global Competency Framework (GbCF) [10], with the importance and relevance of these groupings depending on the individual sectors pharmacists were working in [9].

The majority of pharmacists work in community pharmacies (57%), hospitals (19%), and drug distribution outlets or drugstores (10%). For pharmacy school graduates, the figures are 36% in community pharmacies, 23% in hospitals, and 11% in drug distribution outlets or drugstores [8]. These indicate the one-in-ten pharmacists are now working in drugstores as drugstore pharmacists. Pharmacists working in drugstores (member companies of Japan Association of Chain Drug Stores [11]) were defined as drugstore pharmacists in this survey. Pharmacists in community pharmacies in Japan are mostly focused on dispensing to patients with prescriptions, but those in drugstores not only fill prescriptions but also sell over-the-counter (OTC) drugs as well as a wide variety of health-related goods to people with health concerns. There is a report that about 20% of Japanese people aged 40–74 years had low back pain and knee pain because of the traditional Japanese lifestyle [12]. It seems to be unique to Japan that they sometimes use a fixing tape for reducing temporary musculoskeletal pain and fixing tapes are sold in most drugstores.

As a working environment for pharmacists, drugstores are quite different from community pharmacies. Therefore, professions of drugstore pharmacists do not exactly match with those of community pharmacists and the type of CE programs or training desired by drugstore pharmacists may be different from those needed by community pharmacists. Since Hasumoto et al. indicated that lifelong education of Japanese community pharmacists is still insufficient [13], CE programs for community pharmacists and hospital pharmacists are mostly provided by JPALS and the Japanese Society of Hospital Pharmacists, respectively [8,14]. However, there are few specific organizations providing CE programs for drugstore pharmacists.

There are some reports on future perspectives for CE and CPD, indicating that further development of CE and CPD is still required [15]. Needs-based education is required to develop pharmacists’ education, training, and career development [16] and the competence (knowledge, skills, and attitudes) required to engage in meaningful CPD practice should be introduced and developed at an early stage [17]. These findings suggest CE and CPD programs should be designed at least in part according to pharmacists’ specific working environments.

To develop CE and CPD programs for specialized drugstore pharmacists, it is necessary to identify what they need or want to learn about in CE programs or training. However, research into foundation-level drugstore pharmacists’ perspectives on CE training and CPD programs in Japan has been rare. Therefore, in this study, we asked foundation-level drugstore pharmacists about CE programs and training and what they wanted to learn to provide better pharmaceutical care and customer services. We also discuss the issue of better CE or CPD programs for these pharmacists.

## 2. Design and Methods

### 2.1. Sample

AEON HAPYCOM Comprehensive Personal Training Organization [18] (hereafter AHCPTR) is certified as a provider of the Pharmacist Credentialing Program by the Council of Accredited Pharmacists’ Education Providers [7] and provides CE programs to drugstore pharmacists. Pharmacists who attended the CE program provided by AHCPTR in 2018 were enrolled in this survey. They graduated a 6 year pharmacy education course, and foundation-level pharmacists had only one year of practical experience.

A program organizer explained the purpose of this survey. Then he/she also explained that participation in the survey was completely voluntary and anonymized responses might subsequently be used for publications or presentations. Only those willing to participate in the survey (checked the agree to participate box) were asked to participate in the survey and fill up and hand in the forms. Given that all the relevant factors were fully explained and that participation was stressed to be voluntary, taking part in the survey and handing in form were deemed to constitute informed consent.

### 2.2. Study Design

No control, cross-sectional study.

### 2.3. Survey

At the end of the CE program, the participants were asked to evaluate the overall programs with an evaluation form. In the evaluation form, they were requested to answer an additional question: What kind of CE program, course, or training do you need to develop patient care and customers satisfaction in the future? This was an exploratory survey to refer to future CE programs for drugstore pharmacists.

We used a free-format sheet and some pharmacists filled in multiple ideas. The responses were summarized and collated to identify the products, goods, or services about which the pharmacists wanted to learn in the CE program. Where responses described a special population, they were summarized separately. Each of the various topics mentioned in an individual response was counted separately. For example, a response mentioning “proper use of OTC medicine” and “supplements for pregnant women” was counted under both “OTC medicine” and “supplements” in products and “pregnant women” in special populations.

Data collection was carried out completely anonymously, there was no physical interaction with the participants, and there were no conflicts of interest. In light of these three factors, the criteria laid down by the ethics committee of Josai International University indicates this survey does not require review by the ethics committee.

## 3. Results

We obtained 417 opinions in 280 effective responses from 460 pharmacists (male: 245 and female: 215). The number of evaluation form collected was 398 (response rate: 85.6%), and there were no ideas in some of the evaluation forms. The effective response rate was 60.9% (280/460).

Table 1 summarizes the products or goods that foundation-level drugstore pharmacists wanted to learn about in future CE programs.

The products and goods about which drugstore pharmacists wanted to learn covered a wide range. This would depend on the location (e.g., city center/suburb) or the population mix (e.g., percentage of elderly) near the drugstore where pharmacists were working. They wanted to learn not only about medicines (external medicines or OTC drugs) but also taping skills, tests, and products and devices related to care of the elderly.

The item mentioned most often was knowledge of and techniques for taping and the use of aids and devices for reducing musculoskeletal pain. This indicates that drugstore pharmacists were being asked about taping techniques and the use of such aids and devices very often. Training on the selection and use of external medicines for minor injuries or treatment of minor ailments was almost as frequently requested.

Another topic frequently mentioned was that of tests, especially pregnancy tests. Pharmacists wish to learn about pregnancy/ovulation test kits and related information, including how to use the kits and evaluate the results, suggesting frequent requests for advice by drugstore customers on how to check for pregnancy. Regarding OTC medicines, they wanted to learn how these differ in strength or effect and how to select the best product for customers. There were also some comments regarding the safety of OTC medicines for pregnant women. The interest in learning more about products and devices related to the care of the elderly is clearly related to the significant and ongoing increase in the proportion of elderly people in the population and the pharmacists’ consequent need to know more about this issue.

As health consciousness increases, many Japanese people have become interested in maintaining a healthy life through the use of supplements and health foods. Therefore, knowledge of health foods would be necessary to drugstore pharmacists. The pharmacists also mentioned Kanpo (traditional) medicine as a topic they wanted to learn more about.

There were 67 opinions (67 responses) with any description for the special population of patients (23.9% of 280 effective responses). Table 2 shows the specific populations mentioned by foundation-level drugstore pharmacists. The results indicate that pharmacists want to learn more about safe medication for pregnant and lactating women, and children.

## 4. Discussion

As shown in Table 1, the CE programs or training requested by foundation-level drugstore pharmacists covered a wide area and the item relating to taping and/or aids and devices for helping to reduce musculoskeletal pain would be unique to drugstore pharmacists. Pharmacists’ requests for training in this area would be due to the fact that they are frequently asked to help people with musculoskeletal pain with taping techniques. Many Japanese people are known to complain of musculoskeletal pain in their knees, shoulders, or back. This is probably quite unique to Japan because some musculoskeletal pain (especially knee pain) would be caused by Japan’s “seiza-sitting” culture. Seiza-sitting is characterized by flexing the knees to a high degree, with the feet turned sole upward underneath the buttocks and the tibia internally rotated. Seiza-sitting for long periods is known to cause knee pain and the rates of people with low back pain and knee pain are as high as 20.9% and 18.3%, respectively, for Japanese people aged 40–74 years [12].

Japanese people with musculoskeletal pain often use elastic adhesive tape (i.e., Kinesio tape) to reduce some kinds of musculoskeletal pains in their knee, shoulder, or back. Research into the use of Kinesio tape has not supported its use in clinical practice [19], but it does appear to assist in increasing joint mobilization [20]. People with musculoskeletal pain may frequently ask drugstore pharmacists to apply the elastic adhesive tape because the tape is sold in drugstores, and as healthcare professionals, the pharmacists would be expected to provide information for taping techniques. This is why foundation-level drugstore pharmacists wanted to receive training or education in the appropriate use of elastic adhesive tape. This skill is unique to drugstore pharmacists because Kinesio tape and the devices for reducing musculoskeletal pain are not available at most community pharmacies.

Drugstore pharmacists were found to need information regarding pregnancy and related knowledge (e.g., pregnancy/ovulation tests) and they identified pregnant and lactating women as special populations about whom they needed to learn more. These women tend not to take medication to avoid unnecessary risks, and if they have to take medication, advice from health professionals on the risks of such medication is necessary. These results indicate that drugstore pharmacists, as the most recognizable healthcare professionals in the community, feel knowledge and skills related to women’s health issues, such as the safe use of medication and pregnancy tests, are necessary. Of course, the need for knowledge of women’s health is not limited to drugstore pharmacists but is required for all pharmacists. CE and CPD programs or courses on women’s health, including sexual and reproductive health, are urgently needed for all Japanese pharmacists.

As health consciousness increases, many Japanese people have become interested in supplements and health foods to help maintain a healthy life. Health foods in Japan generally refer to “foods with health claims” and have characteristics between those of a drug and a conventional food [21]. There are two categories, “foods with nutrient function claims” and “foods for specified health uses.” The former contain nutritional supplements (vitamins and/or minerals). Health foods can be sold as foods for specified health uses but not against disease. Patients sometimes over-evaluate the efficacy of health foods against diseases and symptoms, seeing them as similar to drugs. Drugstore pharmacists therefore need the appropriate knowledge about health foods to provide correct information to customers, which is why they want to learn about supplements and health foods in future CE programs.

Pain management is recognized as a cluster of pharmaceutical public health issues in the Global Competency Framework [10], but the necessity for knowledge of taping techniques or related skills to relieve pain was unique to drugstore pharmacists in Japan. This indicates that CE or CPD programs should be developed to meet not only social requirements but also individual pharmacists’ requirements because it was found that learning needs foster pharmacists in their future professional roles [22].

The learning needs of community pharmacists in each country would be different because each country has its own cultural and social background, for example, physical assessment, interpreting laboratory tests, and making decisions about complex drug therapy in Canada [22], leadership, management, and research in Scotland, U.K. [23] and communication, counseling, drug information, and health promotion skills in Qatar [24]. Japanese pharmacists would have their own learning needs.

In this report, we found that foundation-level drugstore pharmacists have CE and CPD requirements that differ significantly from those of community pharmacists. The number of responses was 280, which is not low; however, they were all recruited from participants of the CE program provided by the AHCPTR. This means it is necessary to exercise care when generalizing the findings in this report.

## 5. Conclusions

While pharmacists working in drugstores and similar retail environments constitute a minority of pharmacists worldwide, they are nevertheless a significant section of this profession. The importance of CE and CPD for pharmacists is universally recognized, but the specific needs of this particular group have not generally been acknowledged. The results of this study clearly show that pharmacists in this working environment have unique CE and CPD requirements. The requirements would differ significantly from those of other community pharmacists. The need for specially designed courses to meet their needs is clear.

AHCPTR has already started to provide new CE programs for addressing the issues of taping, women’s health, and health foods based on findings in this research. The outcome of new programs will be reported elsewhere.

## Figures and Tables

**Table 1:** Products or goods about which foundation-level drugstore pharmacists wanted to learn.

Products	No (%) of Responses (*n* = 280)	Examples of Specific Comments
Taping goods/aids and devices (for musculoskeletal pains)	49 (17.5)	Effective taping techniques
External medicines	47 (16.7)	How to select the products and apply to wounds, insect bites, skin diseases
Testers or test kits	42 (15.0)	How to use test kits (pregnancy/ovulation test, blood sugar test) and evaluate the results
OTC * medicine	39 (13.9)	Selection of cold medicines, difference in ingredients depending on the products, interactions with supplements, safe use by pregnant and lactating women
Care of the elderly	33 (11.8)	Characteristics of adult diapers, walking sticks, etc.
Supplements	30 (10.7)	Effectiveness for specific diseases or symptoms
Health foods	26 (9.3)	Effectiveness for specific diseases or symptoms
Kanpo medicine (traditional herbal medicine)	17 (6.1)	Appropriate choice for the patients’ symptoms

* OTC: over the counter.

**Table 2:** Special populations mentioned by foundation-level drugstore pharmacists.

Topics	No (%) of Response (*n* = 67)	Comments
Pregnant women	36 (53.7)	Safe medication, pregnancy/ovulation tests
Children	13 (14.9)	How to take medication (masking of medicines’ bitter taste)
Lactating women	8 (11.9)	Safe medication
